# InSAR data for detection and modelling of overexploitation-induced subsidence: application in the industrial area of Prato (Italy)

**DOI:** 10.1038/s41598-024-67725-z

**Published:** 2024-08-02

**Authors:** Camilla Medici, Matteo Del Soldato, Gabriele Fibbi, Lorenzo Bini, Pierluigi Confuorto, Gaddo Mannori, Alessandra Mucci, Vania Pellegrineschi, Silvia Bianchini, Federico Raspini, Nicola Casagli

**Affiliations:** 1 Earth Sciences Department, University of Firenze, Via La Pira, 4, 50121 Firenze, Italy; 2 Largo San Biagio 149, 51100 Pistoia, Italy; 3 Genio Civile Valdarno Centrale E Tutela Delle Acque, Piazza Della Resistenza 54, 51100 Pistoia, Italy; 4 National Institute of Oceanography and Applied Geophysics - OGS, Borgo Grotta Gigante N. 42/c, Sgonico, Trieste, Italy

**Keywords:** Sentinel-1, InSAR, Field surveys, GBIS model, Subsidence, Tuscany, Environmental sciences, Natural hazards

## Abstract

Spaceborne-based monitoring for environmental purposes has become a well-established practice. The recent progress of synthetic aperture radar (SAR) sensors, including through the European Space Agency’s (ESA) Sentinel-1 constellation, has enabled the scientific community to identify and monitor several geohazards, including subsidence ground deformations. A case study in the Tuscany Region, Italy, highlights the effectiveness of interferometric synthetic aperture radar (InSAR) in detecting abrupt increases in ground deformation rates in an industrial area of Montemurlo municipality. In this case, InSAR data enabled prompt identification of the phenomenon, supporting the authorities in charge of environmental management to thoroughly investigate the situation. First, an on-site validation was performed via field surveys confirming the presence of cracks and fissures on some edifices. Further analysis, including water pumping rates, settlement gauge and topographic levelling, corroborated the InSAR data's findings regarding vertical deformation. Integration of collected data allowed for spatial identification and assessment of the subsidence bowl and its source depth recognized by the remote sensing data. The Montemurlo case offers a procedural guideline for managing abrupt accelerations, identified by InSAR data in subsidence-prone areas due to fluid overexploitation. In fact, these data proved useful in helping local authorities responsible for hydrogeomorphological risk management. With the exacerbation of deformation issues in subsidence-prone regions due to climate change, early detection and monitoring of such phenomena are increasingly crucial, with InSAR data playing a central role in achieving this goal.

## Introduction

Spaceborne-based monitoring for subsidence analysis currently represents a consolidated practice, and several examples can be found in the scientific literature at local and regional scales^1^. The most recent example is that of the EGMS (European Ground Motion Service), which is part of the Copernicus Land Monitoring Service (CLSM)^2^ and provides free access to visualize and download Europe-wide Sentinel-1 data with annual updates. Synthetic aperture radar (SAR) satellite sensors have been systematically used for monitoring the Earth’s surface since the 1990s, and ongoing advancements in SAR satellites and processing algorithms have resulted in several SAR products. The recent growth of SAR sensors comprises the Sentinel-1 constellation, which was developed and launched by the European Space Agency (ESA)^3^; this constellation is formed by two twin satellites, namely, Sentinel-1A (S-1A) and Sentinel-1B (S-1B). However, since December 2021, only S-1A has remained operational for image acquisition due to technical problems, and there are plans to replace S-1B with Sentinel-1C; this constellation provides a systematic supply of images for scientific purposes worldwide^4^. The development of multitemporal interferometry SAR (MTInSAR) processing algorithms^5^, which are strongly connected to satellite enhancement, started in approximately the 2000s^6^, and they are still evolving ongoing evolution. MT-InSAR enables the identification and monitoring of subsidence ground deformation, taking advantage of both displacement velocity and time series (TS) data over time.

Subsidence phenomena are associated with gentle and gradual lowering or sudden sinking of the ground^7,8^. Land subsidence can be related to both natural processes, e.g., the consolidation of soft soils^9,10^, and anthropogenic events, e.g., fluid withdrawal, mining and tunnelling, or carbonate dissolution^11–13^, as well as to their combination^14^. Traditionally, in situ methods such as levelling and global positioning system (GPS) techniques have been used to monitor subsidence phenomena^15–17^. Although these instruments allow reliable and high-precision measurements, they are time-consuming and expensive and do not provide spatially distributed data. These limitations can be overcome with satellite InSAR techniques, a well-established approach for subsidence monitoring over recent decades^1^, enabling measurements with millimetre accuracy, wide area coverage, and relatively low cost. Italy is a territory that is well-known for being affected by natural and anthropogenic subsidence^18,19^, as evidenced by numerous events, such as the natural land subsidence of Ravenna (Emilia–Romagna, northern Italy^20^) or the Sibari Plain (Calabria, southern Italy^21,22^), and the anthropogenic ground subsidence affecting the city of Pistoia^23,24^.

In this work, a case study of anthropogenic land subsidence identified and monitored in an industrial area via MTInSAR data is presented and critically analysed. The results demonstrate how InSAR data can be used for the early detection of land subsidence phenomena and suggest the implementation and adoption of effective land subsidence mitigation policies. This thesis holds particular relevance considering the worsening of climate change affecting our latitudes, which can also impact land subsidence phenomena^25,26^. The Tuscany Region, central Italy, was the first region worldwide, followed by the regions of Valle d’Aosta and Veneto (North–West and North–East Italy, respectively), to provide continuous and operative monitoring, starting in 2016, based on systematic MTInSAR processing every 12 days, i.e., every two new Sentinel-1 radar images. This monitoring has enabled the assessment of trend changes in ground deformation^27,28^. Indeed this approach not only involves the analysis of the ground deformation maps in both geometries but also employs a data mining algorithm on the time series of each point to identify trend variations, i.e., abrupt accelerations or decelerations, called anomalous points (APs)^27^. This monitoring approach runs in the operative direction and has proven to be relevant for the local administrative authorities in charge of environmental management. The authorities are notified by a regular bulletin of the anomalous situations in order to proceed with validating field campaigns^29^. In the framework of Tuscany region monitoring, several critical ground deformation situations, encompassing both landslides^30^ and subsidence^23^, were reported to local authorities in charge of hydrogeomorphological risk management to inform them about the worsening of existing situations or the appearance of newly activated ground deformation scenarios. In addition, the screening of the entire regional territory and the highlighting of critical deformations were useful for better addressing the available funds for restoration or prevention.

**Figure 1:**
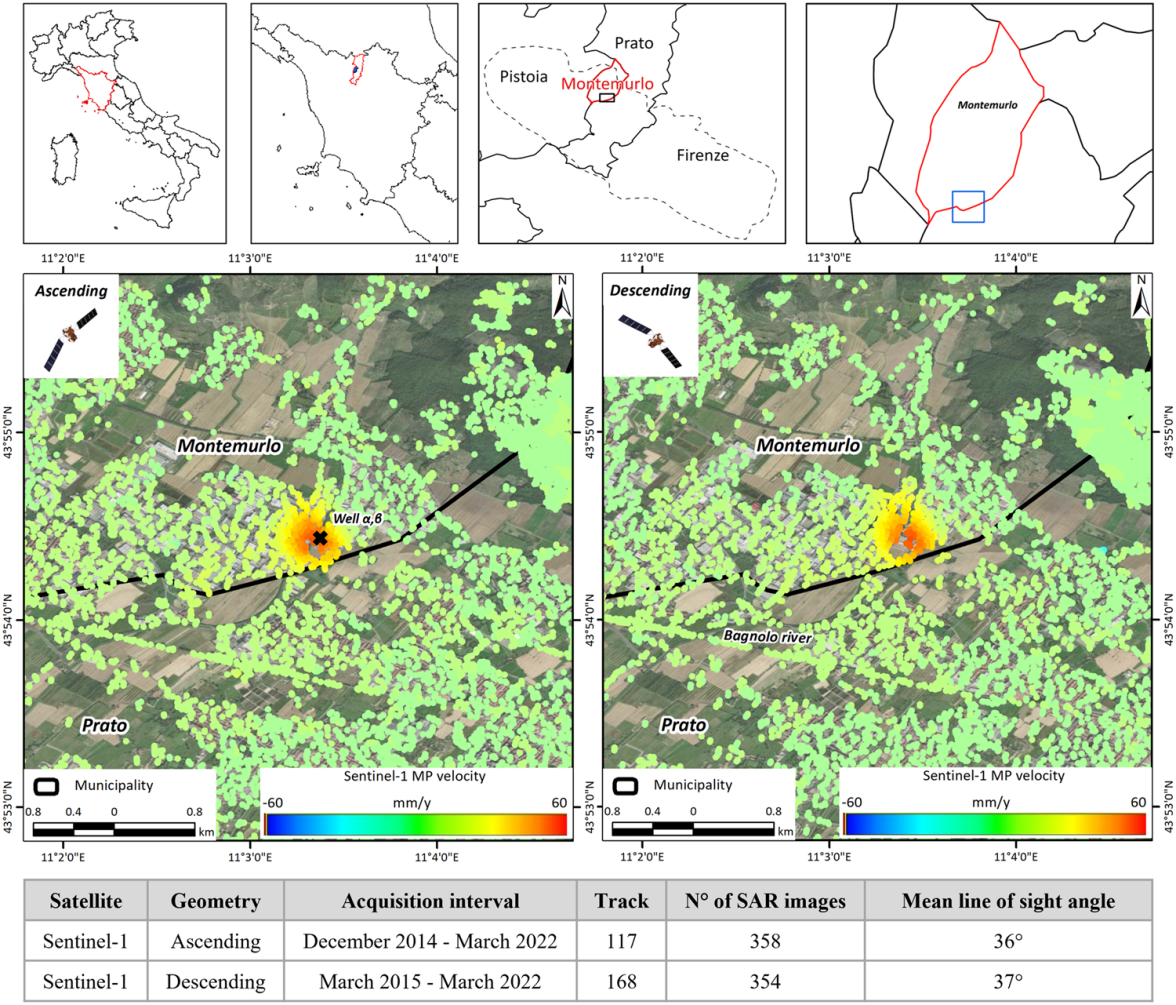
Localization of the area of interest and of the ground deformation. Ground deformation maps of the period 2015–2022 of continuous monitoring in the Tuscany region and characteristics of the Sentinel-1 data.

One of the main relevant subsidence phenomena highlighted by the Sentinel-1 continuous monitoring conducted in the Tuscany Region is the abrupt acceleration recorded in the Montemurlo municipality, Prato Province, in the central sector of the region. The municipality is located on the eastern side of the Firenze–Prato–Pistoia basin, close to the border between the Montemurlo and Prato municipalities (Fig. 1). The area has historically been affected by ground lowering and rebound since the 1990s due to the combination of geological setting^31^ and water pumping for the demand of greenhouses, nurseries and leather factories^32–35^. Geologically, Montemurlo is located on alluvial fan deposits^33^ formed by filling material, and layers of silty and clay material alternating with thin gravel layers; these materials are transported by the Agna and the Bagnolo Rivers, crossing the western reliefs, and deposited on the 150 m-depth Monte Morello and Sillano Formations (Ligurian Domain)^36^.

In recent decades, the industrial district in the southwestern Montemurlo municipality has experienced an increase in manufacturing activities, predominantly in textile production and industrial warehouses, requiring a large amount of water to be pumped from the subsoil. No critical events were recorded before mid-2017.

The continuous monitoring system implemented by the Tuscany Region enabled the identification of a main critical event in July 2017, during which a significant acceleration exceeding -100 mm/year was recorded in the industrial district of Montemurlo. This occurrence was highlighted in both the ascending and descending Sentinel-1 data as shown in Fig. 1 where the characteristics of the Sentinel-1 data are also reported. Once the detected ground deformation was interpreted as real, the authorities in charge of hydrogeological risk management were notified, and further analyses were conducted. Afterward, field surveys for validation and more detailed field campaigns for the detection of damage to nearby structures were conducted. The main aim was to perform further in-depth investigations, identify possible causes, and provide a potential solution for restoring the previous noncritical hydrological scenario. In addition, the depth of the ground deformation source recorded by exploiting MTInSAR data was modelled by using the freely available GBIS (Geodetic Bayesian Inversion Software), commonly used for the modelling of volcanic^37,38^ or seismic events^39,40^ or mining collapses^41,42^ but hereinbefore, to the best of our knowledge, never used for groundwater modelling.

## Results

**Figure 2:**
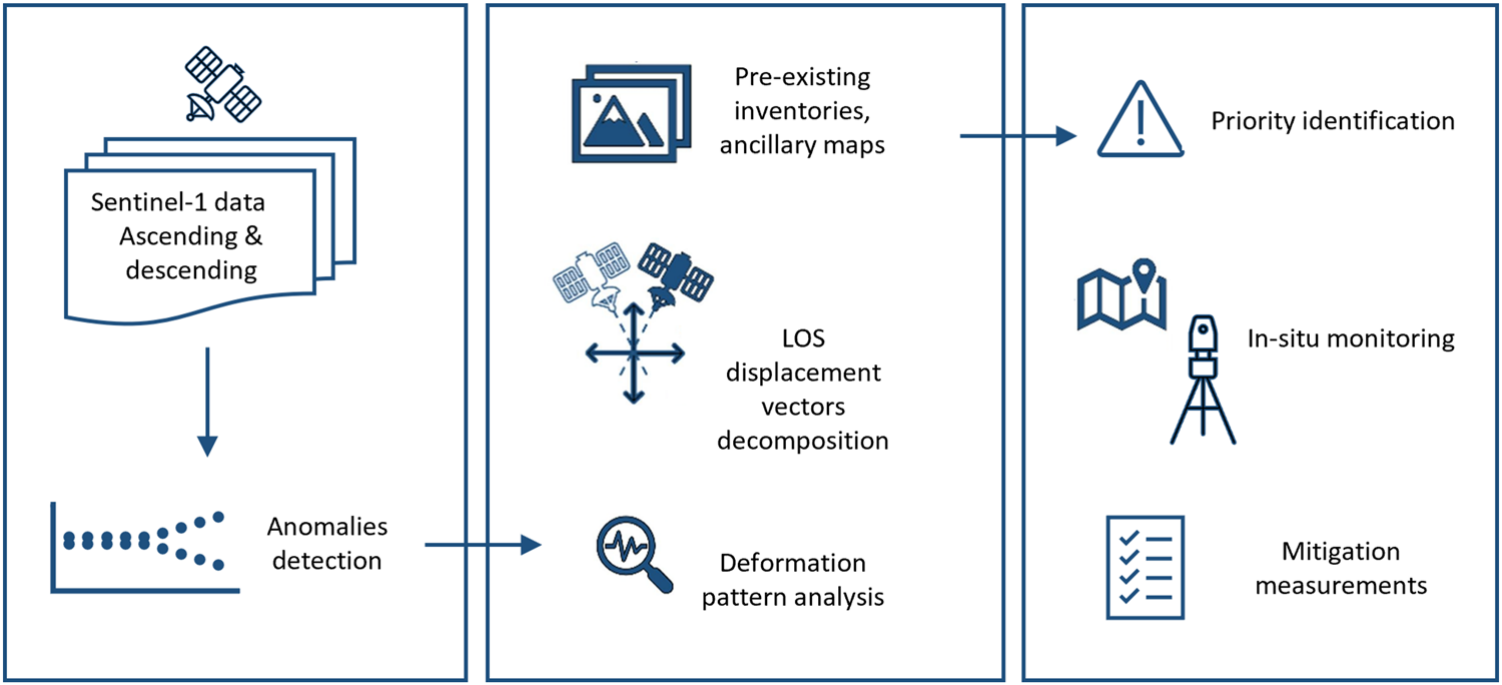
Flowchart for monitoring detected deformation areas by InSAR continuous monitoring.

The main result obtained from the Montemurlo sample case was the successful use of a schematic inductive approach to characterize and monitor the vertical ground deformation recorded by remotely sensed satellite data. In the case of the recognition of moving areas in deformation maps, especially when coupled with anomaly detection, further actions must be taken. First, the usual interpretation and spatial and temporal characterization of the phenomena must be conducted, followed by more in-depth analysis. Once the reality of the recognized phenomena is confirmed, a field survey to determine its effect on structures and infrastructure should be conducted as a campaign for collecting topographic data, e.g., levelling campaigns, global positioning system (GPS) measurements, and systematic total station surveys. In the end, in the case of positive cross-correlation with InSAR data and damage to edifices, which are symptoms of critical situations, further sophisticated instruments, e.g., vertical gauges in boreholes, could be installed to monitor in-depth deformation (Fig. 2). It is very important to characterize the critical ground deformation in detail by combining InSAR data, detecting the effects on the surface, and borehole data to better interpret and understand ongoing phenomena.

The sample case of the industrial area of Montemurlo was adopted to validate the presented approach starting from the spatial and temporal characterization of the phenomena taking into consideration the velocity of deformation and the InSAR time series. By analysing the velocity, it was possible to well-identify the area involved in the deformation. The delimitation can be supported by the decomposition of the velocity in vertical and East–West component facilitating also the characterization of the phenomena. With consideration of the time-series of deformation, and utilizing the mining data analysis for the identification of anomalous points, it was possible to identify the starting moment of the deformation and the temporal evolution, and thus InSAR data with abrupt positive or negative trend variations were obtained. Once the phenomenon was spatial and temporal characterized by remote sensed data, a validation campaign was conducted to cross-correlate the InSAR data with recorded damage by a field survey followed by a levelling campaign, one of the most common techniques of topographic measurement that can be adopted for settlement assessment. At this point, to more thoroughly investigate the critical situation, it was decided that a more sophisticated, precise, and expensive system should be installed: a vertical borehole settlement gauge. This system was useful for (i) collecting geological information of the area of interest, even if strong lateral and vertical variations in short distance are known in this area, and (ii) helping to clarify the vertical deformation in different portions along the column.

### Spatial characterization

**Figure 3:**
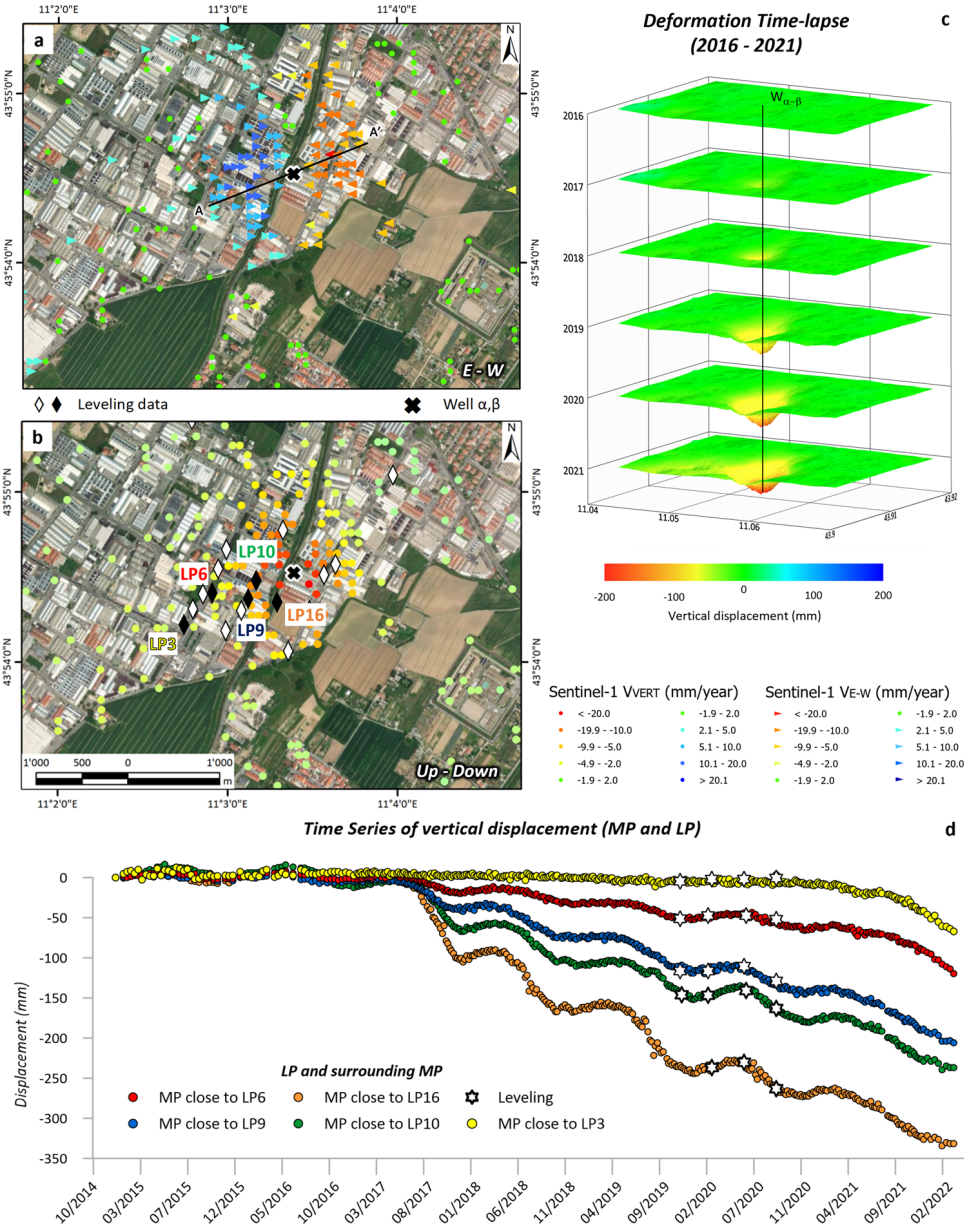
Spatial analysis of the ground deformation of Montemurlo. Ground horizontal (**a**) and vertical (**b**) velocity components of deformation in the industrial area of Montemurlo. Vertical ground deformation maps (× 10 exaggerated) by year (**c**) and time series of measurement points cross-correlated with nearby levelling points (**d**).

The Sentinel-1 InSAR measurement points (MPs) over the area of interest in the Montemurlo municipality show a relevant bowl of subsidence in both ascending and descending orbits (Fig. 1). The correspondence of the ground deformation maps between the MP recorded in ascending and descending geometries confirms the predominance of a vertical motion, as well as an almost circular ground motion bowl, slightly elongated toward the east and north. This information suggests the occurrence of anthropic-induced subsidence. Line of Sight of the satellite (LoS) velocities higher than 20 mm/year were recorded over an area of approximately 200,000 m^2^ (0.2 Km^2^), which becomes 400,000 m^2^ (0.4 Km^2^) considering velocities greater than 10 mm/year. A maximum LoS velocity of approximately 50 mm/year in both ascending and descending orbits was registered on both hydrographic sides of the Bagnolo River. The data are even more evident and easier to understand by analysing the east‒west (Fig. 3a) and vertical (Fig. 3b) velocity components. Interestingly, the horizontal component, moving from east to west, initially exhibited a prevalent east component, followed by a predominant west component immediately to the left of the Bagnolo River. The vertical component, instead, shows a radial inward increment. Considering the directions of the two velocity components, the resulting shape seems to develop a cone face down (Fig. 3b), a shape usually associated with an overpumping effect. The velocity shapes of the LoS and the E‒W and Up–Down components are also relevant for identifying potential sources of subsidence. Considering the position of the MP with the highest velocities, the possible source area is localised in correspondence with an industrial area and, more precisely, near the wells denoted as W_α-β_, which are employed by an industrial laundry (Fig. 3b).

The evolution of vertical deformation (exaggerated 10 times for easier visualisation) affecting the ground over the year from 2016 to 2021 (Fig. 3c) confirms that in 2016, the deformation was negligible, and it became significant in 2017 after an abrupt acceleration. In the following years, the stability of the system was compromised, and the deformation continued to increase.

The time series of the vertical velocity component registered in five MPs localised near the topographic levelling points (LPs) were analysed. In this context, starting from the east (LP6) and progressing to the west (LP16), a sudden increase is observed approaching the Bagnolo River, where the W_α-β_ wells are situated. The good correspondence between the InSAR and levelling time series of point 3 demonstrates that the recorded deformation is localized close to that area and that no regional trends affect this area. By comparing the TS of the vertical component acquired through the MTInSAR and those obtained through the levelling campaign, a perfect overlap of the measurements can be observed (Fig. 3d); this can be seen, in particular, in the time span November 2019–August 2020, during which four measurements were carried out in a network of benchmarks (Fig. 1).

### Temporal characterization

The Tuscany Region, Central Italy, in 2016 was the first region worldwide to implement continuous monitoring through the SAR system, based on Sentinel-1 data and time series data mining, performed every 12 days (and thus every two new Sentinel-1 acquisitions). The service highlights the MPs affected by an acceleration or deceleration greater than 10 mm/year in a temporal window of 150 days^27^. The MPs identified according to the abovementioned procedure are defined as anomalous points (APs). In 2020, continuous monitoring was limited to a 3 month update (systematic) while maintaining the same alerting procedure.

**Figure 4:**
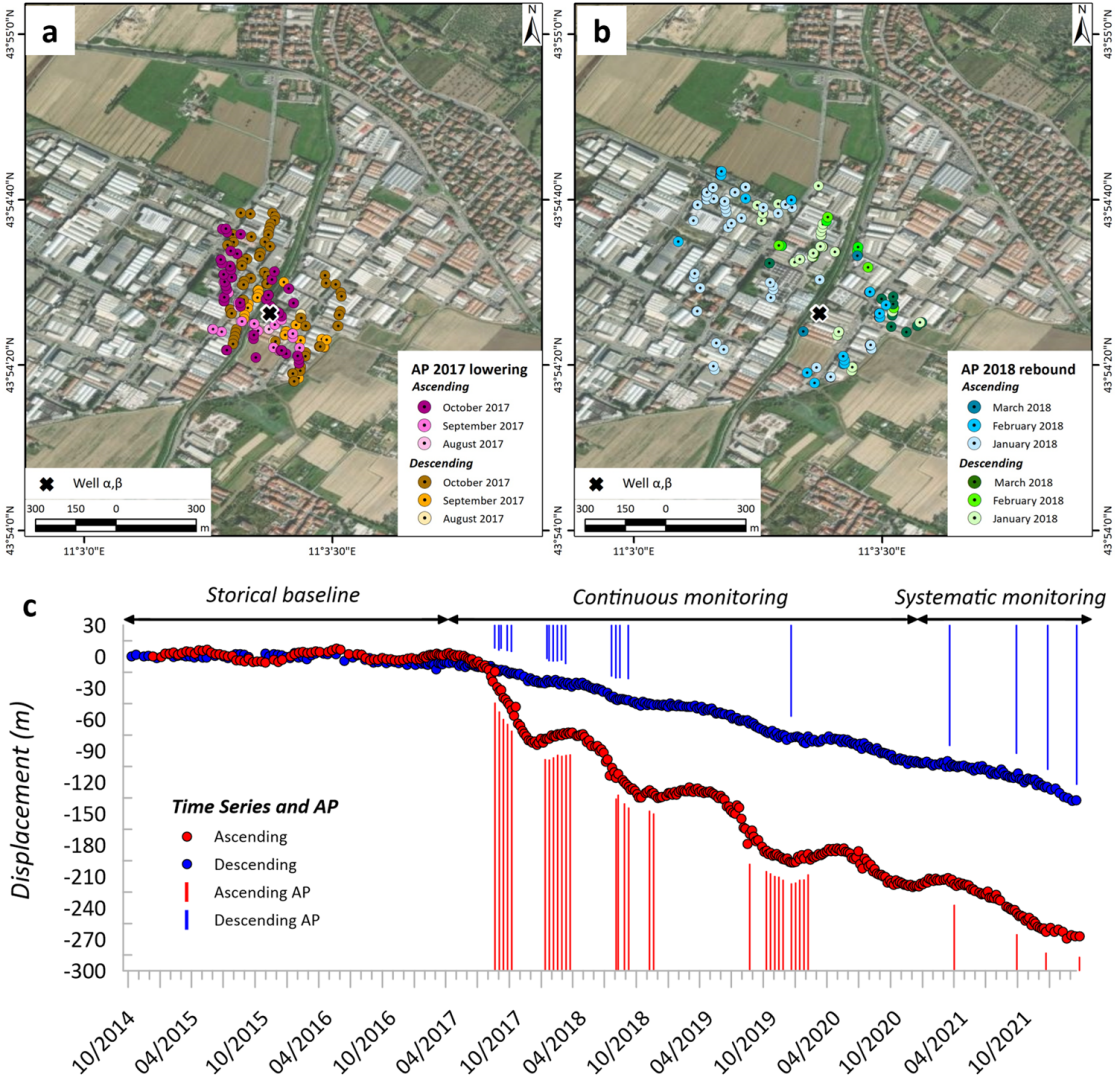
Spatial (**a**) and temporal distribution of anomalies in the area of interest. Spatial localization of the MPs showing acceleration or deceleration in the ascending or descending (APs) orbits and temporal distribution compared with a time series per orbit.

In the industrial district of Montemurlo, the first APs were recorded in August 2017, both in ascending and descending geometries, due to an abrupt acceleration in the time series. It is worth emphasizing that from October 2014 until July 2017, the velocity ranged around the stability threshold of ± 2 mm/year. From August to October 2017, the number of updates showing APs increased, and the area involved in the lowering increased. Thus, from January to March 2018, an elastoplastic rebound was recorded in both acquisition geometries, involving a slightly larger area. Interestingly, the spatial distribution of the temporal occurrence of the AP demonstrates the evolution of ground deformation over time. Notably, the anomalies in both the ascending and descending orbits started in August 2017 and continued to expand from the source outward until October 2018, forming an elongated N‒S shape (Fig. 4a). From January 2018, the anomalies in both geometries were compared for the rebound effect, and they were automatically identified until March 2018, starting from the furthest portion with respect to the well to the source area (Fig. 4b).

In 2018 and 2019, during approximately the same period of the year, a similar situation was recorded, mainly in the ascending geometry, even if the acceleration was lower than that in 2017 (Fig. 4c). Since 2020, there has been a decrease in the number of updates showing anomalies in ground lowering and rebound in both ascending and descending geometries. This decrease is a direct consequence of the reduced number of updates.

### Design of in situ monitoring

**Figure 5:**
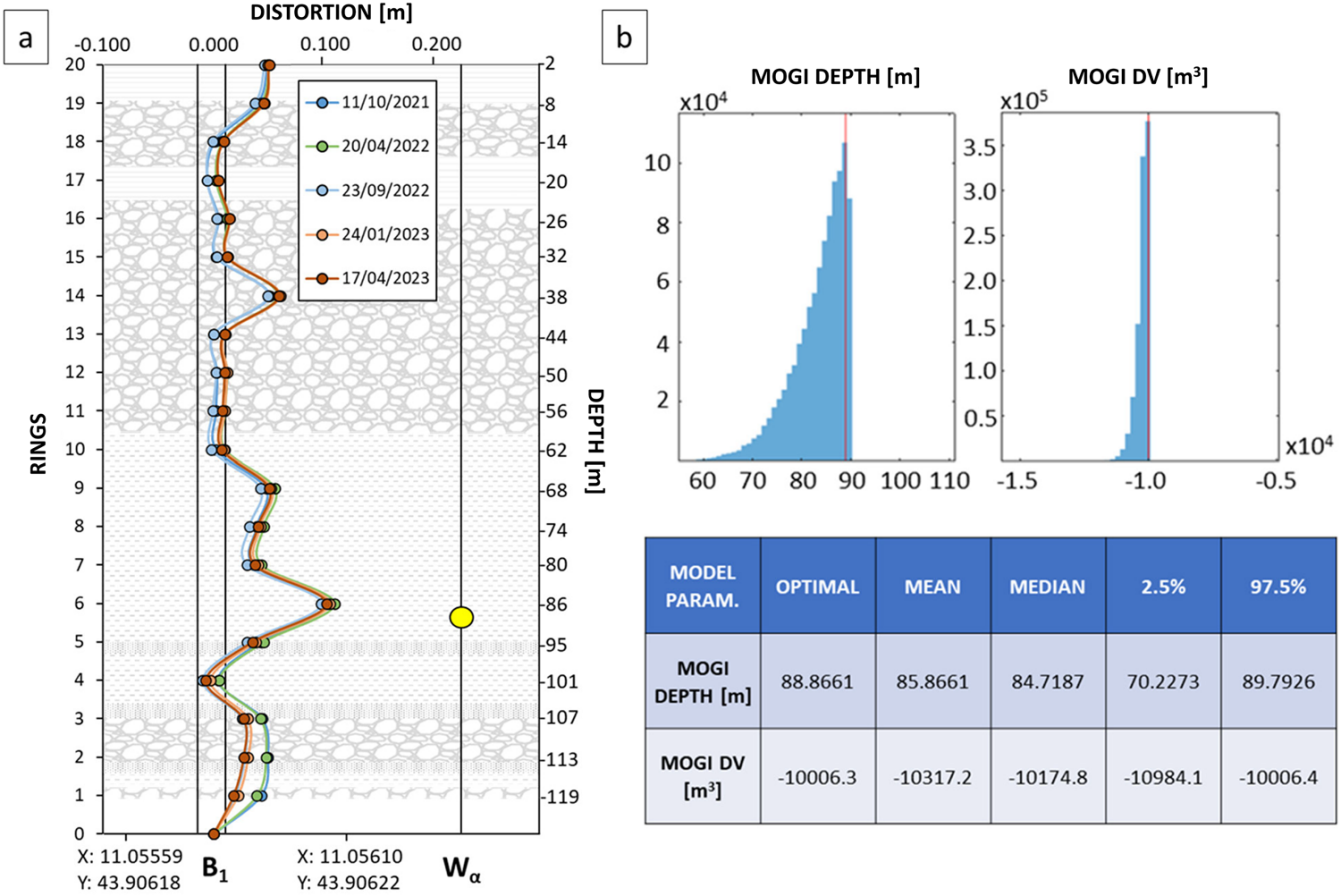
Vertical distortion close to the Wa measured by the vertical gauge in the borehole (**a**) and GBIS assessed results of depth and volume changes (**b**).

In the sample case study, several measurement campaigns using a topographical levelling instrument were conducted. In addition to the low number of measurements and frequency, the MP and LP differential displacements between subsequent measurement results are coherent, and the correlation between them is clear, confirming the ongoing phenomenon. Then, considering the continuous lowering of the ground, a more precise and sophisticated instrument, a vertical gauge, was installed close to the possible source of the deformation. The measurements were collected approximately every 12 days, after an initial settling period during which the data seemed poorly correlated, providing valuable insights for understanding and monitoring the phenomenon. Significant displacements over time were observed in two portions of the boreholes, specifically at rings 14 and 6, which correspond to depths of approximately 38 and 86 m, respectively (Fig. 5a). In the upper section, the recorded displacement can be neglected since it could be caused by several external factors, and this displacement cannot be related to the ground deformation detected by the MTInSAR data. Interestingly, the displacement recorded at a depth of 38 m corresponds to that of sand and gravel, with millimetric to centimetric and occasionally decimetric dimensions within a silty-clayey matrix. However, the lower deformation, close to 86 m depth, corresponds to a clayey silt layer with locally abundant gravel characterized by millimetric to centimetric grains.

### Ground deformation causes

As mentioned above, the area identified as the subsidence source was very close to the laundry pumping wells. To confirm the hypothesis that the subsidence might be due to groundwater exploitation by the laundry, the pumping and water level data of two wells, the Laundry α, which has a depth of 140 m, and the Laundry β, which is more superficial, located in the laundry area at a distance of approximately 1 m, were collected six times from October 2019 to October 2021 (Table 1). In addition, the entity of water withdrawn and the depth of the water level were also collected.

**Table 1:** Pumping data of the two wells (Laundry α and β) close to the identified source area of ground deformation.

Data	Well	Pumped water (m^3^)	Water level (m)
26 September 2019	Laundry α	52,892	24.87
	Laundry β	1527	10.48
09 March 2020	Laundry α	–	–
	Laundry β	–	–
25 June 2020	Laundry α	64,892	22.60
	Laundry β	1527	11.30
23 October 2020	Laundry α	76,308	29.40
	Laundry β	1527	9.37
16 April 2021	Laundry α	83,560	12.20
	Laundry β	1527	5.50
13 October 2021	Laundry α	99,887	38.50
	Laundry β	1527	8.40

According to the combination of the recorded pumping rate and the ground deformation maps obtained from MTInSAR and levelling data, the water withdrawal from the Laundry α could be the cause of the subsidence bowl. The complex geological setting of the area, composed of alluvial sediments with several intercalations and small lenses with higher permeability, can lead to strong and rapid deformation in a well-defined area due to the rapid depletion of the lenses and consequent drainage of water into the surrounding less permeable portions. Unfortunately, no pumping or water table depth information from previous years was available for comparison with the recorded MTInSAR data reporting abrupt acceleration.

It is worth noting that from September 2019 to October 2021, pumping was only conducted in Laundry α (approximately 25 km^3^ per year), but the changes in the groundwater level depth were strongly recorded in both wells, with decreases of approximately 27.2 m and 6.7 m for Laundry α and β, respectively. The nonproportional responses of the water level changes in the two closed wells can be attributed to the different depths of the wells and the distinct aquifers tapped and to the strong complexity of the geological setting of the area, despite their proximity.

### GBIS model of the deformation source

The GBIS model^43^ allows for assessing the source depth of deformation and estimating the volume change based on the velocity and extent of MTInSAR data. In this case, the GBIS was applied to determine the depth and assess the volume variation in the subsidence resulting from excessive groundwater resource exploitation. After more than 10^6^ iterations, the GBIS model placed the subsidence bowl at a depth of approximately 88 m. The assessed depth corresponds to a deep layer composed of clayey silt with gravel, characterized by a permeability of 6.6 10^−9^ m/s and uncertain lateral continuity. Notably, the permeability at this depth is greater than that in the lithology just below (95 m depth), confirming that water could be extracted at this depth. To estimate the source depth, a point pressure source model^44^, as presented by Mogi, representing embedded deformations in a uniform elastic half-space with Poisson’s ratio ʋ = 0.25, was considered. The adopted model also accounts for the deformed volume, the affected area and the displacement velocity provided by the MTInSAR data in both the ascending and descending geometries. The volume changes were assessed by calculating the cubic metres of soil affected by subsidence. A conical volume calculated considering the height equal to the total deformation achieved in the borehole area and a radius assessed considering a minimum deformation velocity of 20 mm/year (between 200 and 650 m, resulting in 8373 m^3^ and 88,443 m^3^, respectively) were considered. These large ranges of values were chosen to avoid forcing the model results. The outcomes show an optimal volume change of approximately − 10,006 m^3^ and a mean volume change of approximately − 10,317 m^3^ (Fig. 5b).

### Multiplatform data combination

**Figure 6:**
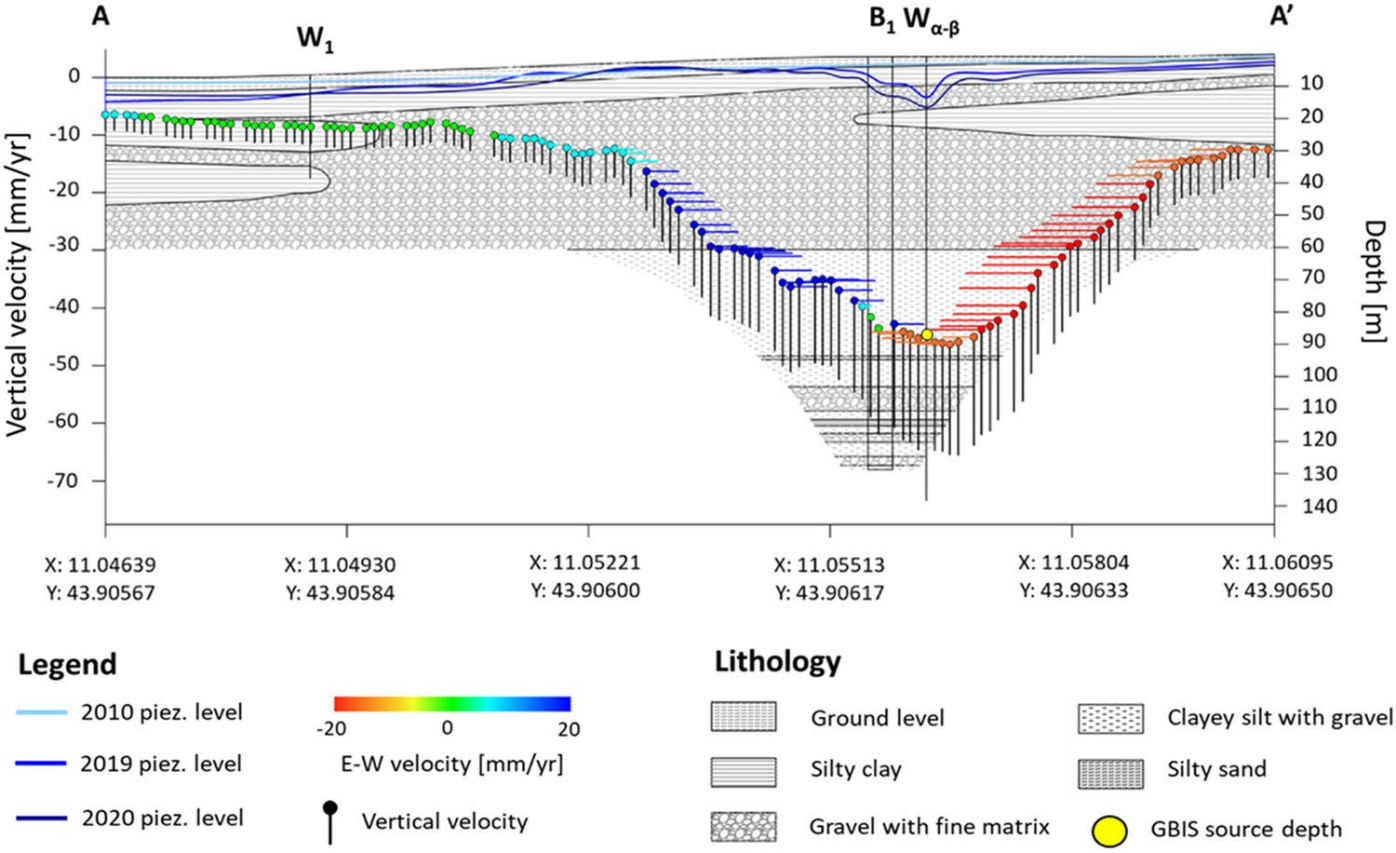
Section A-A’ (Fig. 3a), including all the involved subsidence analysis data and the GBIS-assessed source depth of the area of interest. The section shows the piezometric levels at different times, i.e., 2010 (pre-event), and 2019 and 2020 (post-event), on the geological background and the vertical and horizontal components recorded by the MPs.

A comprehensive analysis including all available and collected data was carried out to understand the possible causes and their effects. Section A–A’ of the area of interest (Fig. 6) shows all the collected subsidence analysis data and the source depth assessed by the MOGI model in correspondence with the Laundry well α with a good approximation by the use of the GBIS tool. The section (Fig. 6) crosses the area of interest (Fig. 3a), intercepting two available boreholes with known geology and the one drilled in 2018 for the geological investigation of the area, successively equipped with a vertical settlement gauge. In the background, the stratigraphic setting (modified from Capecchi et al.^31^) has been inserted for an easy comprehension of the lithological formation in which the GBIS model suggests that the deformation source is located, specifically the clayey silt with gravel (Fig. 6). The graph presents the piezometric levels at different times, i.e., 2010 (pre-event, in light blue), 2019 and 2020 (post-event, in blue and in dark blue, respectively), and the vertical and horizontal components of the velocity obtained by MTInSAR. The combination of all the data allows easy identification of a direct correlation between the vertical ground deformation and the piezometric deflection collected after 2017. In fact, the maximum vertical displacement results aligned with the lower piezometric level, specifically where well α of the industrial laundry is located. In addition, a perfect symmetry of the horizontal component of the deformation velocity shaping a pumping cone centred on the Wα can be recognized, confirming the hypothesis that this area is the source of the subsidence induced by the high-rate groundwater extraction. Further confirmation is provided by the vertical settlement gauge, which shows greater deformation at a very close depth to ring number 6, registering the vertical deformation at a depth of 86 m below the surface (Fig. 6).

## Discussion

The integration of multiplatform and omni-comprehensive monitoring data allows the characterization of subsidence events and helps in understanding the phenomena that occurred. In fact, MTInSAR data can be used to identify the evolution of deformation and the timing of the beginning of the event, to define a possible source area, and to delimit the affected area. In 2018, Raspini et al.^27^ showed the first worldwide application of the MTInSAR technique for monitoring a wide area, an entire Italian region, by using a time series data mining approach to identify ground deformations affecting the territory early. Although the applicability and efficacy of this method for landslide detection have already been demonstrated^30^, the present work provides good operability for subsidence phenomena. Moreover, the abrupt acceleration of the Montemurlo area has been identified owing to the continuous update of the MTInSAR data for the area, consisting of both ground deformation maps and data mining for highlighting the MPs with changes in their trends. Continuous monitoring allows the early identification of phenomena with the possibility of alerting personnel in charge of hydrogeological and territory management, with the final aim of preventing hydrogeological compromise or damage to structures and infrastructure.

The Tuscany Region planned to establish this system to develop an operative chain^29^ that could be activated in the case of abrupt acceleration in areas where consequences could occur. In the subsidence of the Montemurlo industrial area, the operative chain was activated in a very short time, after just two MTInSAR updates, where an abrupt acceleration in both ascending and descending data was recognized within an industrial area. In recognition of the potential consequences of the recorded displacement, a field survey was promptly conducted within a few days to validate and clarify the entities and their impact on structures and infrastructures. In the end, the necessary actions, such as pumping rate limitations, further investigations, and adoption of monitoring systems, were identified. In fact, in the case of abrupt acceleration in subsidence-prone areas where several wells pump groundwater are active, a rapid mobilization to elucidate and characterize the phenomenon is fundamental to avoid operative consequences during nearby activities.

The approach adopted in the Montemurlo case study should be replicated in every subsidence-prone area. Considering the automatic classification conducted by the InSAR data, the Italian territory has more than 300 areas classified as subsidence and more as potential subsidence^19^ due to different causes: (i) continued pumping in known water-stressed basins, causing water depletion and consequent lowering of ground level^45^; (ii) coastal subsidence due to overpumping^46^, with possible critical consequences such as seawater intrusion or for volcanic systems^47–49^; and (iii) natural consolidation of sediments or overload for rapid urbanization in soft soils or subsoils^50^. In addition, subsidence is more commonly caused by load imposition, mining activity and hydrocarbon extraction^20^, sinkhole formation resulting from karst dissolution^51^, geothermal exploitation^52,53^, permafrost^54,55^, land reclamation^56^ and dumping and landfill^57,58^. Moreover, almost all these causes can be exacerbated by the ongoing climate changes of recent decades, with implications for groundwater levels and aquifer recharge^25,59^. In fact, drought periods are becoming more prolonged, while rainfall is becoming increasingly concentrated, both temporally and spatially, causing relevant changes in groundwater dynamics. In urbanized coastal areas, the risk compounded by climate change is that overpumping of coastal groundwater resources causes salinization^60^ as a consequence of the reduced value of the aquifer and its impoverishment as a source of freshwater. The contamination of coastal freshwater affects not only the water quality for the water supply of coastal communities and their economic development but also vegetation and animal health, as well as the environment in coastal areas^61,62^.

Enlarging the MTInSAR investigation over Europe, subsidence-prone areas and zones already affected by subsidence phenomena can be recognized by using the European Ground Motion Service (EGMS) data of the Copernicus Land Monitoring Service (CLMS), e.g., the Yela Underground Gas Storage (UGS) site^63^ can be monitored by a systematic yearly update^64^. Considering the global perspective on subsidence issues, more than a thousand scientific contributions covering the period of 1971–2021 were collected and critically analysed by Raspini et al.^1^, demonstrating the high impact and interest regarding these phenomena worldwide.

As stated by Bagheri–Gavkosh et al.^26^, subsidence phenomena have multiple implications, e.g., socioeconomic, environmental, and protection issues^65,66^, mainly in urbanized areas close to deltas^67^. Therefore, the development of an investigative and operative approach can improve the possibility of avoiding catastrophic events or depletion of the groundwater source. The added value of the presented approach lies in the possibility of applying MTInSAR monitoring with annual updates and investigating the source of the phenomenon, and no additional costs are associated with the use of EGMS data. The information that can be extracted from this MTInSAR screening and the time series data mining allows for (i) early highlighting of stable areas beginning to show ground deformation, (ii) supporting the decision in territory management, (iii) prioritizing the actions to take place, starting from more critical and urgent situations, and (iv) scheduling accurate adjustment works supporting territory planning at diverse levels. This work represents a starting point for areas where MTInSAR monitoring, levelling campaigns or the installation of further instruments, e.g., GNSS receivers or vertical settlement gauges, could be beneficial. The possibility of customizing the analysis to meet the specific requirements is an advantage of this approach rather than a drawback. In fact, throughout Europe, the identification of subsiding sites, their probable causes, and possible 2D sources could be hypothesised at very low costs due to the large amount of free data available from Copernicus services and the use of GBIS open-source software, which was first tested in this work for a subsidence case study. The Mogi model offers a simplified representation of the deformation source. Despite its simplifications, the elastic model provides valuable insights into the deformation patterns observed in the InSAR data. The elastic deformation model has been chosen also in view of a semi-elastic behaviour of the deformation (see Fig. 4c). The subsiding trends followed by an uplift phase may be due to poroelastic rebound mechanism in the multi-layered aquifer system. This pattern is triggered by piezometric level rise due to pumping interruption. The heterogeneity of geological layers and their complex geotechnical properties (e.g. viscoelastic or plastic deformation, and poroelastic effect) are not accounted in the simple eleastic model. As a consequence, this may lead to discrepancies between the modelled and actual deformation. To overcome these limitations, in situ information were incorporated for obtaining more accurate and reliable results. The results were satisfactory, and such an inductive approach can support the authorities in charge of hydrogeological risk and management to be systematically updated over possible critical situations and to foresee further studies, e.g., geological insights or additional or more frequent MTInSAR analyses and actions, e.g., the installations of cutting-edge instruments, as presented in this work.

## Methods

The data presented in this study for the investigation of this exemplary case of groundwater pumping-induced subsidence are derived from different monitoring and processing approaches, which are briefly explained below.

### Sentinel-1 interferometric products

The MPs (measurement points) data are derived from the systematic Multi-temporal interferometric synthetic aperture radar (MTInSAR) processing of Sentinel-1 radar images by the SqueeSAR algorithm^68^, an evolution of the PSInSAR algorithm^6^. In addition, the Sentinel-1 MPs were further automatically managed by a data mining algorithm that works on the time series and allows the selection of only MPs that show an abrupt acceleration or deceleration greater than 10 mm/year in a defined period of 150 days^27^. These parameters were defined in the first year of testing for the Tuscany Region, considering the possible effects of ground deformations on structures and infrastructure, as well as on the different environments across the territory. The MPs data were decomposed into east–west and up-down components^69^ to better identify and analyse the subsidence effects and enable comparisons with levelling measurements.

### Multitemporal piezometric level reconstruction

The temporal evolution of the piezometric curves was reconstructed considering the measurement of the water level depth with respect to the ground, which was conducted twice per year, specifically during the spring, a period of lean water, and during the autumn, a period of soft water, in 2019 and 2020. Thirty measurement points covering an area of approximately 3.1 km^2^ were used to reconstruct the phreatic level of the area via IDW (inverse distance weight)^70,71^. The use of the same spatialization approach allows comparison of the resulting surface, extraction of relevant information about the temporal changes, and identification of possible causes, considering other factors, e.g., water withdrawal. In addition, a piezometric level dataset from 2010 was used to compare the situation before the critical events of 2017.

### Topographic levelling data

The topographic surveys were conducted four times from November 2019 to September 2020, yielding millimetric precision dependent on the point distance and instrument used^72^. Sixteen measurement points were collected within a restricted area to monitor the area of interest and cross-correlate the displacement recorded by the remote sensing data, i.e., the MPs data derived from the Sentinel-1 radar images.

### Vertical settlement gauge

In 2019, a deep borehole was drilled at the location of the maximum displacement recorded by the MTInSAR data, in which a magnetic vertical settlement gauge was installed. This instrument consists of a double guide tube, an external tube formed by a flexible corrugated tube, and an internal tube in polyvinyl chloride (PVC) around which magnetic rings are connected to the surrounding ground. In the Montemurlo case study, 20 rings were placed every 6 m. The magnetic properties of these rings allow for the monitoring of vertical settlement between pairs of rings; data are collected by inserting a sensor into the tube for measuring the position of each ring with respect to the borehole’s bottom. These measurements are relative; in fact, the first one is useful for calibrating the “zero” to which all the future measurements will be referred.

### Combination of all data for causes identification

All the available data were combined and compared to determine the cause of the identified ground subsidence. The vertical component of the MPs was combined with the pumping data and the reconstructed water table, and this combination allowed us to investigate the possible causes of ground subsidence. All the data were plotted on a graph to highlight the influence of pumping on the ground and the water table.

### GBIS model

The inversion of the mean LoS (line of sight) displacement rates obtained from the time series analyses is useful for estimating the parameters of a contracting source at the depth that best aligns the SAR observations^73^. This method allows for determining the source depth and its possible relationship with anthropogenic activities. The open-source geodetic Bayesian inversion software (GBIS)^43^ was used to perform the inversion through ascending and descending displacement rate images from the Sentinel-1 satellite data. This software was developed primarily for retrieving volcanic sources and active fault geometry^74,75^, but in this study, it was adapted for anthropogenic deformation. The predictive analytical model chosen to estimate volume changes due to fluid poroelastic effects is the point source model^44^. The volume change limits were determined by calculating the cubic metres of soil affected by subsidence. A conical volume was calculated using a height equal to the total deformation observed in the well area and a radius evaluated at a minimum deformation rate of 20 mm/year. This ranged between 200 and 650 m, resulting in volumes of 8373 m^3^ and 88,443 m^3^ respectively. In addition, a maximum depth of 90 m was considered, corresponding to the limit depth in the strata exploited by the well. In order to ensure the most accurate and reliable results, the limits of the modelling intervals were carefully chosen based on in-situ measurements. A multidisciplinary approach, specifically incorporating settlement data, was adopted to achieve robust quality control.

## Data Availability

The data that support the findings of this study are available from the corresponding author upon reasonable request.
